# Contentment

**Published:** 1892-06

**Authors:** J. A. Robinson

**Affiliations:** Jackson, Mich.


### Contentment.

Dedicated to my Friends who have seen Eighty Years.

Not old nor lame, nor halt nor blind,
But well preserved in frame and mind;
My hearing is a little tough,
Perhaps I've really heard enough.

My home is pleasant, clean and neat,
The inmates lovely, kind and sweet;
With comforts rare my table's spread,
My sleep is sound, and soft my bed.

Of this world's goods no more I crave,
If they will last me to the grave.
The great beyond will soon be here,
The path is peaceful, vision clear.

You ask me why I do not sigh?
It is because we never die.
This world's a link in endless chain,
We decompose to live again:

Where we our olden friends will meet,
When the gestation is complete.
You ask me how that fact I know?
My reason says it must be so:

For God who made the world and plan,
And made it for the home of man,
Did not make man for gain or pelf,
But in the image of himself.

With heart and soul with love to burn,
And love his Maker in return.
He could not leave the work undone,
And so he sent a perfect Son

To show how it would take effect,
And in the end would resurrect.
So Christ appeared at early dawn;
Both Marys saw him in the morn.

The angel rolled the stone away
Just at the dawning of the day;
A symbol of the rising sun
That the new life was now begun.

His angel robes shone as the light,
His face shone with effulgence bright;
He told the Marys "not to fear"
"For Christ has risen—is not here."

The Marys then believed the word
That they should see the risen Lord.
At Emmaus he appeared again
And talked with his two chosen men.

He left the two men in the street,
And met eleven who sat at meat;
They did not recognize his face
Nor know him 'till he said the grace.

Through all the Gospels and the Acts
Of the Apostles are these facts.
In ancient history of Rome
We learn that various acts were done;

We may not get the truth exact
But still have record of the fact.
Macaulay's England tells of fact
That may not always be exact.

Plutarch's history of lives and men
Show they were great and not in vain;
The record of the very fact
Perhaps is not the most exact,

But still we have the distant view
And we believe the record true.
The evidence is very small
Compared with what is told by Paul.

Damascus was the chosen place
Where Saul would meet Christ face to face,
An enemy intent to slay
The Saviour on a certain day.

Christ met him with a shining light
Before he had a chance to fight
And said: "Why persecute thou me?"
The light was bright; Saul could not see,

But said: "Who is it that I hear?"
The answer came, Saul shook with fear,
He knew the voice; he could not see;
It said: "Why persecute thou me?"

As if to make it still more plain,
The Christ gave Saul his real name.
This record written out by Saul
After his name was changed to Paul

Is fact and no legend or fable,
And told by a man amply able
To give the facts he heard and saw,
Without a blemish or a flaw.

This record, if it stood alone,
Will stand a million years to come,
As it has stood in all the past,
And will not fail while time shall last.

The book has been revised again
By scholars and by learned men.
No one who reads the story through
Can fail to see it must be true.

He who in life would lead the van
Must always be an honest man;
He must be brave, he must be right
To be a hero in the fight;

Arkwright was hero of the spinner,
And Paul the hero of the sinner;
He never let his labor cease,
But followed Christ, the Prince of Peace;

Watts gave the steam the world to free,
And Franklin electricity.
Lincoln was hero in freedom's cause,
And Grant to execute the laws;

All were a part of nature's plan
To raise and elevate the man.
The protoplasm that we know,
The lowest form begun below,
Is just as sacred in its place
As man is to complete the race.

From center to circumference I think it must be true
That life's reaction always has a higher life in view;
The granite rock, the mineral, was first to re-appear;
Next came vegetable life, and that not very near.

Through earthquakes and commotion, upheaval and turmoil,
The granite rock was crushed and broke to make alluvial soil;
Next came vegetable life, the very lowest form,
Warmed by the sun and wet with rain for moss to grow upon.

Thro' years of change and waiting long the higher grasses came,
And then the reptiles, then the birds, the order is the same;
So all along these million years the higher order ran;
The onward march has never slept till it came up to man;

And so I think you cannot fail to fully see and know
That man retains some vital part of everything below.
We find in man the mineral in tissue and in blood,
Then vegetable comes along the very self-same road.

And then at last the animal, the lowest form of man,
And that must be the order that our human life began.
God's providence is over man and all the things we know,
But man has also providence o'er little things below;

Man's providence is over land, to plough and till the ground,
To beautify God's providence and smaller things around,
To make two spears of grass to grow where one had grown before,
To build the cities and the schools, and help the needy poor,

To take the elements of earth and make them better still,
To make himself and other things obedient to God's will.
God's kingdom, if it comes at all, must come thro' human hands;
We all must work together as we beautify the lands;

Our duty and our privilege is to press along while here,
And when the other life shall come we nothing need to fear.
If it is true that man retains a portion of the past,
The finished life will lead him to the heavenly life at last.

As birds look up with little eyes the mother birds to see,
So we by constant looking up can see Infinity.
Now, my old friends of four score years, say, how is it with you?
Have I shown you the great beyond, and made the picture true?

Does the new world we're going to your hungry souls invite,
And way across the valley dark do you behold the light?
If you do not, go meditate upon the heavenly day,
Listen to hear the heavenly voice, and work and watch and pray.

JACKSON, MICH., May 31, 1892. J. A. ROBINSON.

